# PET OWNERSHIP AND LONGITUDINAL CHANGE IN PSYCHOLOGICAL ADAPTATION – EVIDENCE FROM THE BLSA

**DOI:** 10.1093/geroni/igad104.1401

**Published:** 2023-12-21

**Authors:** Erika Friedmann, Nancy Gee, Eleanor Simonsick, Barbara Resnick, Merve Gurlu, Ikmat Adesanya, Soyeon Shim

**Affiliations:** University of Maryland, Baltimore, Maryland, United States; Virginia Commonwealth University, Richmond, Virginia, United States; NIA IRP, Baltimore, Maryland, United States; University of Maryland, Baltimore, Maryland, United States; University of Maryland, Baltimore, Baltimore, Maryland, United States; University of Maryland Baltimore, Baltimore, Maryland, United States; University of Maryland Baltimore, Baltimore, Maryland, United States

## Abstract

Successful aging depends in part on maintaining psychological adaptation. Research examining the relationship of pet ownership (PO) or human-animal interaction (HAI) to human health supports contributions to psychological status, mostly in populations with diseases or disabilities. We examine the contributions of PO to maintaining psychological adaptation among generally healthy community-dwelling older participants in the Baltimore Longitudinal Study of Aging (BLSA). Participants (N=637, age M=68.3, SD=9.6 years, pet owners N=149) completed assessments of anxiety (Perceived Stress Scale-10), depression (Center for Epidemiological Studies Depression), happiness (1-10 rating) and psychological wellbeing (SF-12 Mental Component Score) every 1-4 years and a 10-year PO history. Linear mixed models with random intercepts were used to examine changes in psychological adaptation outcomes over up to 13 years (M=7.5, SD=3.6) according to time-varying PO. Overall anxiety decreased (b=-0.0360,se=0.0104,p=0.001) and depression increased (b=.099141, se=0.015837, p< 0.001) with aging. Psychological wellbeing (b=0.00055, se=0.00119, p=0.644) and happiness (b=-.00405,se=0.0206,p=0.844) did not change. PO moderated changes in anxiety (b=0.058452,se=0.024376,p=0.017), happiness (b=-0.101373,se=0.04779,p=0.034), and psychological wellbeing (b=-0.005541,se=0.002764,p=0.045), but not depression (b=-0.01134, se=.037065,p=0.769). Anxiety and happiness improved in pet owners and deteriorated in non-owners. Psychological wellbeing deteriorated faster for pet owners than non-owners. This study provides important longitudinal evidence that PO may promote some aspects of psychological adaptation among generally healthy community-dwelling older adults by moderating age-related declines in psychological adaptation later in life. It also highlights differences in psychological responses to animal assisted interventions in residents of care facilities from changes during the life-courses of generally healthy community-residing older adults.

